# Social isolation, loneliness and the relationship with serum biomarkers, functional parameters and mortality in older adults

**DOI:** 10.1007/s40520-025-03041-4

**Published:** 2025-05-03

**Authors:** Stefanie Braig, Michael D. Denkinger, Dhayana Dallmeier, Jochen Klenk, Dietrich Rothenbacher, for the ActiFE Study Group

**Affiliations:** 1 Institute of Epidemiology and Medical Biometry, Ulm University, Helmholtzstraße 22, 89081 Ulm, Germany; 2 Research Unit on Ageing, Geriatric Center Ulm/Alb-Donau, AGAPLESION Bethesda Clinic Ulm, Ulm, Germany; 3 Institute for Geriatric Research, Ulm University Medical Center, Ulm, Germany; 4 Department of Epidemiology, Boston University School of Public Health, Boston, MA USA; 5 Department of Clinical Gerontology, Robert Bosch Hospital, Stuttgart, Germany

**Keywords:** Biomarkers, Cardiac markers, Loneliness, Mortality, Social isolation

## Abstract

**Background:**

Pathways between social isolation (SI), loneliness and health are unclear.

**Aims:**

To analyze the relationship between SI and loneliness with biomarkers of inflammation, cardiac and immune function, functional parameters, and mortality.

**Methods:**

SI (Lubben Social Network Scale) from family, friends, and overall as well as loneliness (single direct question) were assessed at baseline in a population-based cohort study of 1459 community-dwelling adults aged 65 + in Germany. Serum biomarkers and functional parameters measured at baseline and at three-year follow-up included high-sensitivity C reactive protein (hs-CRP), growth differentiation factor-15 (GDF-15), N-terminal pro-brain natriuretic peptide (NT-proBNP), high-sensitivity troponin I (hs-cTnI), high-sensitivity troponin T (hs-cTnT), gait speed, and hand grip strength. We used linear and Cox regression analyses adjusted for age and sex (model 1) and established confounders (model 2).

**Results:**

High SI from friends was associated with small but significant adverse associations with some biomarkers (hs-CRP, GDF-15, hs-cTnT) at follow-up (model 1). High SI from family associated with NT-proBNP (model 2), high SI and moderate to severe loneliness with lower gait speed. Loneliness was linked to hs-CRP at baseline, but SI was a stronger predictor of biomarker levels. High SI overall (Hazard ratio 1.39, 95% CI 1.15; 1.67, model 2) was associated with increased 10-year mortality.

**Discussion:**

Mainly SI from friends is linked to unfavorable biomarker profiles with small associations. Overall SI was negatively associated with functional parameters and positively with mortality.

**Conclusions:**

Further research should confirm our findings using, e.g. a multidimensional assessment of loneliness.

**Supplementary Information:**

The online version contains supplementary material available at 10.1007/s40520-025-03041-4.

## Introduction

Loneliness is prevalent in industrialized countries with the highest burden among young and older adults [1], but results are mixed across age groups [2] and geographic regions [3]. According to a recent meta-analysis, the pooled prevalence of loneliness among older adults in high-income countries was around 30% [4]. While loneliness refers to the subjective feeling of a discrepancy between one’s desired and actual level of social contact, social isolation (SI) describes the objective absence of relationships [5]. It is estimated that 24% of community–dwelling adults aged 65 + in the United States are socially isolated with 4% experiencing severe social isolation [6]. In a US study with participants aged 60 + the prevalence for loneliness was 43.2% [7].

Health consequences of SI and loneliness are becoming increasingly recognized [8, 9]. A meta-analysis of prospective studies showed that SI or loneliness were associated with increased risks of incident coronary heart diseases and stroke [10]. The association between SI and an elevated risk of heart failure was modified by loneliness [11]. Systematic reviews further suggest inverse associations of SI and loneliness with mental health [12] with less strong evidence for physical than mental health outcomes [13]. Nonetheless, both of them are related to all-cause mortality [9, 14–16].

The mechanisms that might explain the link between SI, loneliness and health are not fully understood. Chronic social stress is associated with an enhanced sympathetic and altered hypothalamic–pituitary–adrenocortical axis activity [17, 18] leading to pro-inflammatory monocyte expansion and cytokine expression. However, their involvement in SI and loneliness needs further verification [19]. Associations between biological markers such as the growth differentiation factor-15 (GDF-15), which is involved in inflammatory and apoptotic pathways, and SI or loneliness are sparse, even though GDF-15 has been linked to mental health symptoms [20]. There is also a lack of research on the association between SI and loneliness and N-terminal pro-brain natriuretic peptide (NT-proBNP), a cardiac marker of left ventricular function in particular, which has been associated to emotional and mental health conditions [21, 22]. Besides, cross-sectionally, a poor social network was negatively associated with mean gait speed [23], while longitudinal results are inconsistent [24–26].

The aim of the study was to investigate the cross-sectional and longitudinal relationship of SI and loneliness with i) inflammatory, cardiac, and other well-established blood-based biomarkers, ii) functional parameters, and iii) the 10-year mortality in community dwelling older adults in order to explore potential clues for involvement of well-known inflammatory, cardiovascular, and functional markers.

## Methods

### Study population

**Fig. 1:**
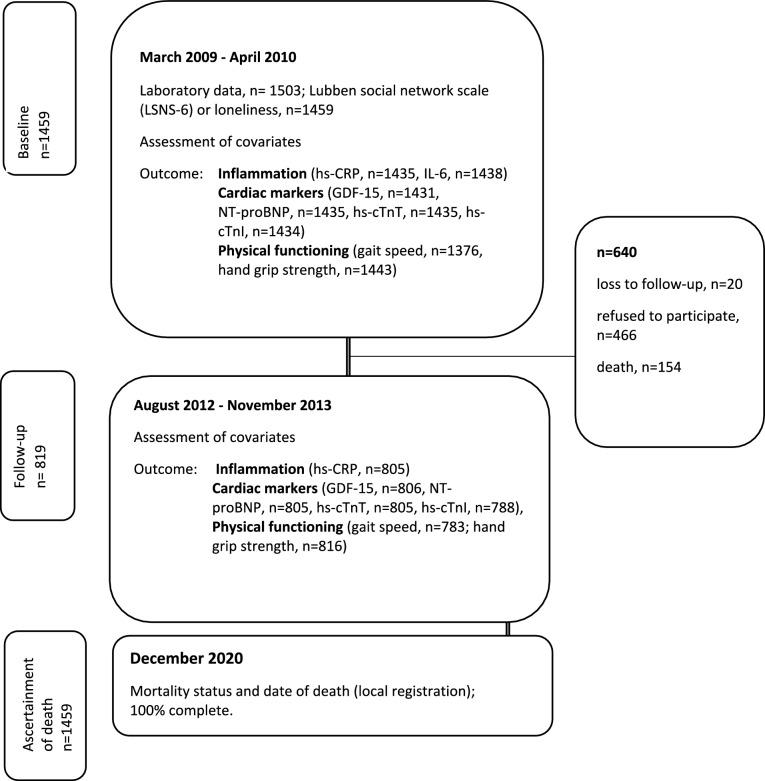
Flow chart of study participants from baseline to follow-up

The Activity and Function in the Elderly in Ulm (ActiFE Ulm) study is a population-based cohort study in subjects aged 65 + years at baseline, randomly selected from Ulm and adjacent regions (Southern Germany). A standardized assessment was completed by trained research assistants. Exclusion criteria were: being in residential care or having serious German language difficulties. Persons with severe deficits in cognition were not included as they were not able to give informed consent. The baseline study took place between March 2009 and April 2010. N = 1506 older people agreed to participate and underwent detailed assessment (initial response rate = 20%). N = 1503 provided biomarkers. The cohort and the measurements taken have been previously described [27, 28]. The three-year follow-up (FU) study took place between August 2012 and November 2013 including 853 (57%) participants. The study population consists of 1459 participants providing data on SI and loneliness and blood samples at baseline and 819 at FU (see Fig. 1). All participants gave written informed consent. The Ethics Committee of Ulm University had approved the study (application no. 318/08 and 50/12).

### Social isolation (SI)

SI from friends and neighbours and SI from the family were assessed using the two subscales of the Lubben Social Network Scale (LSNS-6). The LSNS-6 is a six-item scale that measures levels of SI using the number and frequency of social contacts and social support among the family (3 items) and friends/neighbours (3 items). In our study, the scales were inverted, i.e. high scores correspond to high self-reported SI. We calculated the cumulative score of SI from family and friends/neighbours (SI overall). For correlations, the scales were used continuously, while for further analyses, they were categorized at > 6 points (non-inverted scales for family or friends/neighbours) or > 12 points for SI overall [29].

### Loneliness

Loneliness was assessed by a single question: *“Sometimes one can feel lonely even if it doesn’t appear that way, because you may have many relatives. How lonely do you feel given a scale from 0 (not at all) to 10 (totally)?”* For analyses other than correlations, the variable was categorized as follows: None (0 points), mild (1–3 points), and moderate to severe (4–10 points).

### Social isolation and loneliness

To assess the importance of SI versus loneliness, we created a variable that was 0 if participants were neither socially isolated nor lonely, 1 if participants were socially isolated but not lonely (loneliness 0 points), 2 if participants were socially isolated and lonely (≥ 1 point) and 3 if they experienced no SI but loneliness (≥ 1 point).

### Outcomes

During baseline and 3-years FU, blood samples were taken under standardized conditions, centrifuged, aliquoted, and stored at − 80 °C. We measured high-sensitivity C reactive protein (hs-CRP), Interleukin-6 (IL-6), GDF-15, NT-proBNP, high-sensitivity troponin I (hs-cTnI), high-sensitivity troponin T (hs-cTnT), and Cystatin C according to the process suggested by the manufacturers. Interleukin 6 (IL-6) was measured at baseline only. Online Resource (Online Resource 1) provides detailed information on the applied methods.

Additionally, habitual gait speed in m/s (standardized depending on the walking distance) was measured at baseline and at follow-up. So was hand grip strength (kg, JAMAR dynamometer, Sammons Preston, Bolingbrook, Illinois (USA)).

### Ascertainment of deaths

Mortality status and date of death were obtained from the local registration offices for all participants in December 2020, 10 years after baseline. This date was considered as date of censoring.

### Covariates

Age, sex, and highest duration of school education (≤ 10 or > 10 years) as well as living alone (yes/no) originating from the baseline questionnaire were assessed. We asked for marital status (married, single, divorced/separated, widowed, living apart together), smoking (never, former, current), and alcohol consumption (daily, several times per week, > several times per week) at baseline and follow-up. Similarly, we asked for the number of medications used at both time points.

Body height and weight were measured by the interviewer to calculate the body mass index (BMI) in kg/m^2^. We further considered Cystatin C based Glomerular Filtration Rate (GFR, ml/min) at baseline as covariate. However, we did not control for physical activity (PA, daily duration of walking based on a 7-day sensor-based measurement by means of an accelerometer (activPAL, PAL Technologies Ltd., Glasgow, UK)) [28] as we considered PA as mediator. An author of our group showed that SI was associated with lower levels of PA and this predicted more depressive symptoms after 3 years [30]. Second, PA mediated the association between loneliness and CRP in a study of 3735 US respondents [31] and preliminary results showed convincing arguments for mediation in our data (results not shown).

We further considered depressive symptoms assessed by the Hospital Anxiety and Depression Scale (HADS) [32] at baseline and follow-up as mediators of the association between SI or loneliness and health (biomarkers) as argued by e.g. Holt-Lunstad and colleagues [15]. Therefore, we did not adjust for depressive symptoms.

### Statistical analysis

Characteristics of the study population are displayed. We provide a stratified analysis according to participants’ follow-up status. Median biomarker values including interquartile ranges (1st and 3rd quartiles) were calculated, as well as Spearman correlation coefficients with the independent variables adjusted for age and sex. For correlations with the variable “SI and loneliness”, we calculated dummy variables and point serial correlations also adjusted for age and sex.

Using multiple linear regression analyses, we examined the cross-sectional associations and in addition, we analyzed the associations among participants who participated at baseline and FU. Model 1 was adjusted for age and sex, Model 2 additionally for education, living alone, number of medications, BMI, smoking, and alcohol consumption, living alone, and GFR.

Cox proportional hazards models were used to estimate the effect of SI overall and loneliness (categorized) at baseline on 10-year mortality. The proportional hazards assumption was assessed graphically and statistically. For linear regression and Cox proportional hazards models the outcome variables were log-transformed in the presence of skewed distribution especially for hs-CRP. Analyses were performed using SAS 9.4 and R 4.3.2.

## Results

As shown in Table 1**,** mean age of the study population at baseline was 75.6 years (SD = 6.6) and 77.6 years (SD = 6.0) at 3-years FU. At baseline, 342 (23.4%) older adults indicated to be socially isolated from family and 595 (40.8%) from friends. The respective value for SI overall was 390 (26.7%). Conversely, 804 (55.1%) perceived no, 420 (28.8%) mild, and 235 (16.1%) moderate to severe loneliness. Among those 390 socially isolated, 205 (52.6%) felt also lonely, while 185 (47.4%) denied loneliness. Median follow-up time was 10.8 years; during follow-up 527 (36.1%) deceased.

**Table 1:** Description of study population at baseline and follow-up^a^

	Baseline			Follow-up
	Overall n = 1459	Characteristics of subsample with follow-up n = 819	Characteristics of subsample without follow-up n = 640	Characteristics at follow-up n = 819
Age [years], mean (SD) at baseline	75.6 (6.6)	74.2 (6.0)	77.3 (6.8)	77.6 (6.0)
Women, n (%)	638 (43.7%)	338 (41.3%)	300 (46.9%)	–
Education, n (%) ≤ 10 years	821 (56.9%)	428 (52.8%)	393 (62.2%)	–
Marital status at respective time point, n (%)				
Married	945 (65.0%)	559 (68.5%)	386 (60.6%)	545 (66.6%)
Single	57 (3.9%)	37 (4.5%)	20 (3.1%)	31 (3.8%)
Divorced/separated	83 (5.7%)	49 (6.0%)	34 (5.3%)	49 (6.0%)
Widowed	362 (24.9%)	169 (20.7%)	193 (30.3%)	190 (23.2%)
Living apart together	6 (0.4%)	2 (0.3%)	4 (0.6%)	3 (0.4%)
Living alone, n (%)	354 (24.6%)	194 (24.0%)	160 (25.4%)	–
Smoking at baseline, n (%)				
Never	729 (50.0%)	409 (49.9%)	320 (50.0%)	–
Former	625 (42.9%)	357 (43.6%)	268 (41.9%)	–
Current	104 (7.1%)	52 (6.4%)	52 (8.1%)	–
Alcohol consumption at baseline, n (%)				
Daily	440 (30.7%)	254 (31.5%)	186 (29.7%)	–
Several times/week	378 (26.4%)	214 (26.5%)	164 (26.2%)	–
> Several times/week	615 (42.9%)	339 (42.0%)	276 (44.1%)	–
Number of medications used at respective time point, mean (SD)	3.8 (2.8)	3.5 (2.6)	4.2 (3.0)	4.5 (2.9)
BMI [kg/m^2^], mean (SD) at respective time point	27.6 (4.2)	27.5 (4.0)	27.6 (4.4)	27.4 (4.2)
Depression symptoms (HADS-D) [points] at baseline, n (%)				
< 8	1238 (88.9%)	724 (91.8%)	514 (85.1%)	–
8–10	107 (7.7%)	45 (5.7%)	62 (10.3%)	–
> 10	47 (3.4%)	19 (2.4%)	28 (4.6%)	–
GFR [ml/min], mean (SD) at baseline	81.5 (20.2)	84.6 (18.8)	77.9 (21.3)	–
Duration of walking [min], mean (SD) at baseline	104.0 (40.1)	109.8 (38.0)	96.3 (41.5)	–
Social isolation at baseline, n (%)				
From family	342 (23.4%)	173 (21.1%)	169 (26.4%)	–
From friends	595 (40.8%)	298 (36.4%)	297 (46.4%)	–
Overall^b^	390 (26.7%)	177 (21.6%)	213 (33.3%)	–
Self-perception of loneliness at baseline, median [Q1,Q3]	0 [0;3]	0 [0;2]	0 [0;3]	–
Self-perception of loneliness at baseline, n (%)				
None (0 points)	804 (55.1%)	464 (56.7%)	340 (53.1%)	–
Mild (1–3 points)	420 (28.8%)	242 (29.6%)	178 (27.8%)	–
Moderate to severe (4–7 points)	235 (16.1%)	113 (13.8%)	122 (19.1%)	–
Social isolation and loneliness baseline, n (%)				
No SI, no loneliness	619 (42.4%)	382 (46.6%)	237 (37.0%)	–
SI without loneliness (loneliness = none)	185 (12.7%)	82 (10.0%)	103 (16.1%)	–
SI with loneliness (loneliness ≥ mild)	205 (14.1%)	95 (11.6%)	110 (17.2%)	–
No SI but loneliness	450 (30.8%)	260 (31.8%)	190 (29.7%)	–
Median follow-up time (years)				10.8
Number of deceased, n (%)	–	–		527 (36.1%)
Death rate per 1000 person-years	–	–		40.0

*BMI* body mass index, *GFR* cystatin C based glomerular filtration rate [ml/min], *kg* kilogram, *m* meter, *min* minute, *ml* millilitre, *SD* standard deviation

^a^SI from family and friends does not sum up to SI overall

^b^the percentages might differ as a result of missing values

At follow-up, younger and married participants, and adults who took few medications were overrepresented. Additionally, participants who took part in the follow-up study experienced less overall SI as well as SI from friends, with less moderate to severe loneliness.

Measures of central tendency and dispersion are shown in Online Resource 2. Small but statistically significant positive correlations became evident between SI from friends measured at baseline and hs-CRP FU (r = 0.08, p = 0.018), GDF-15 baseline (r = 0.05, p = 0.043), hs-cTnT baseline (r = 0.06, p = 0.028), and hs-cTnT at FU (r = 0.11, p = 0.002). Furthermore, SI and loneliness were negatively correlated with gait speed at baseline and follow-up (z-score) with the highest (negative) correlation for SI overall and gait speed at baseline (r = −0.14, < 0.001). SI from friends and overall was negatively correlated with handgrip strength. Correlation coefficients between SI and loneliness in the subgroup of participants who were followed-up are displayed in Online Resource 3.

**Fig. 2:**
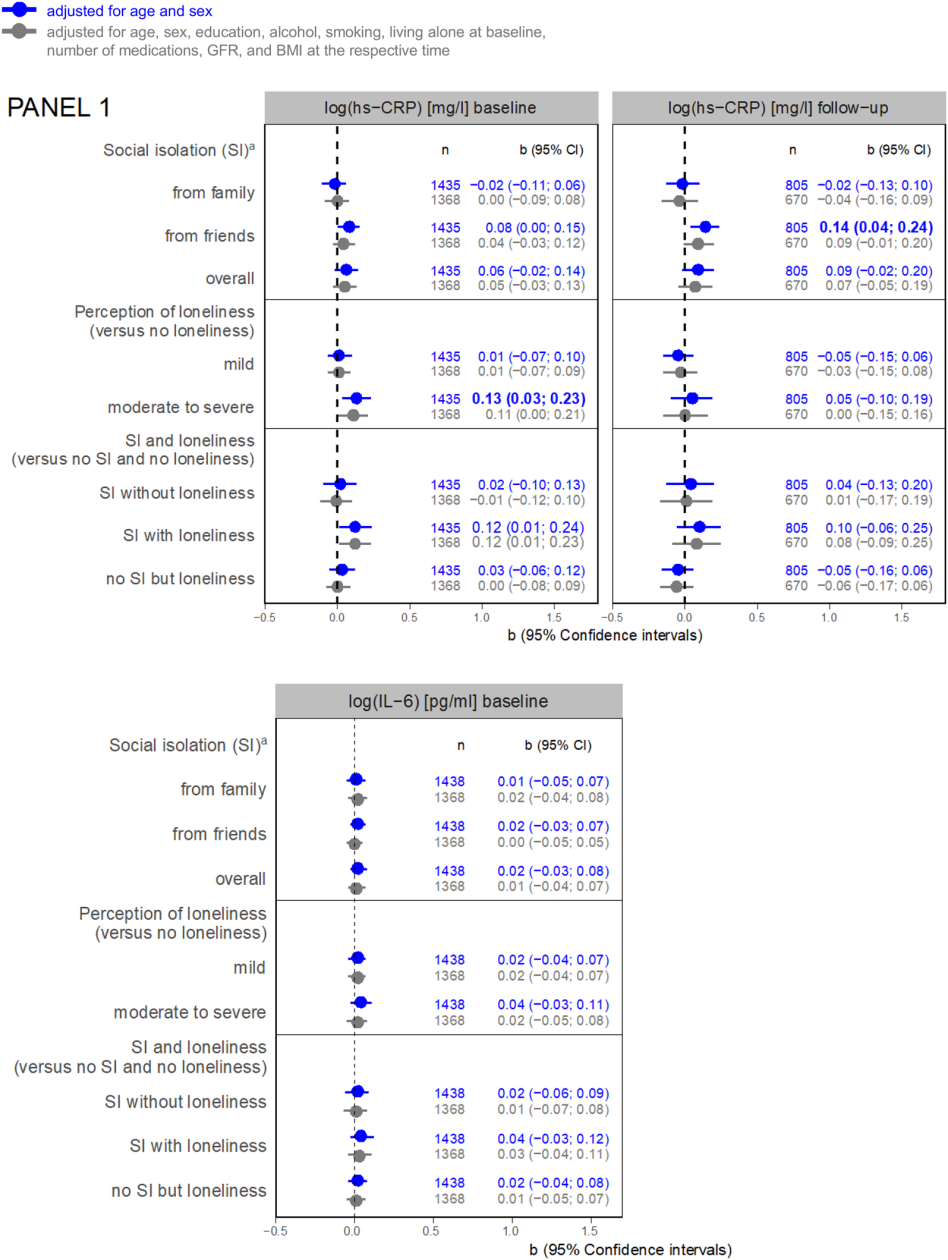

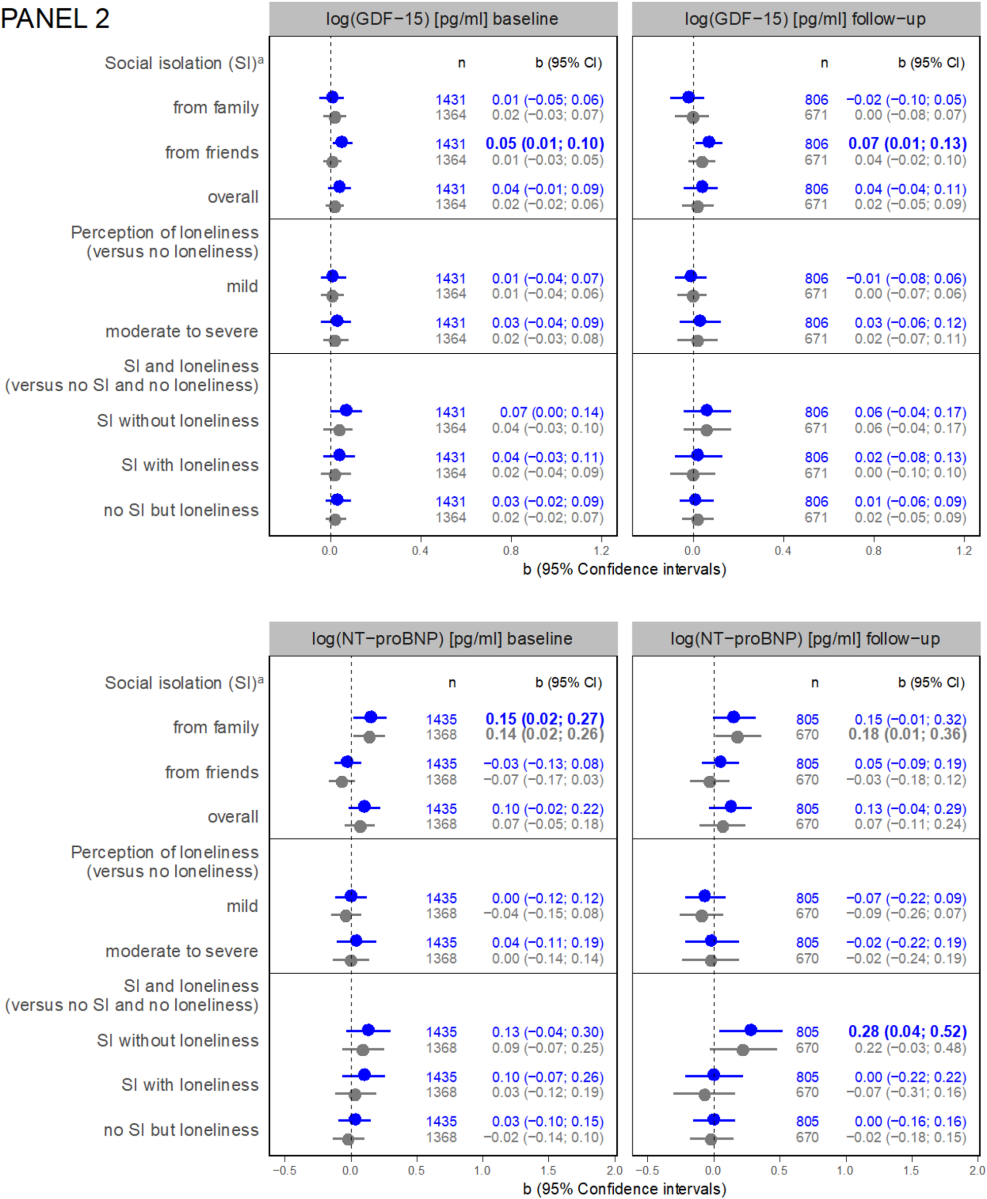

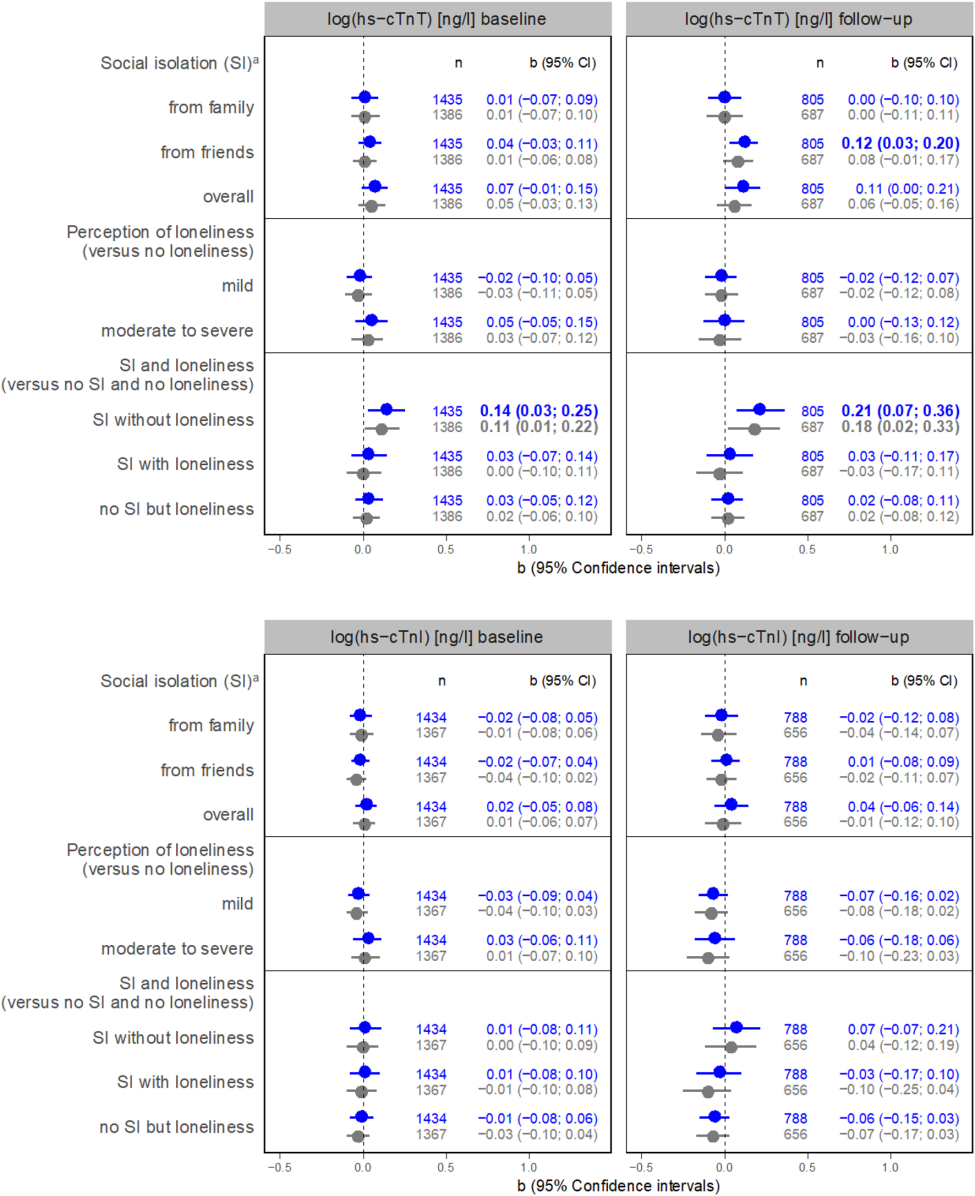

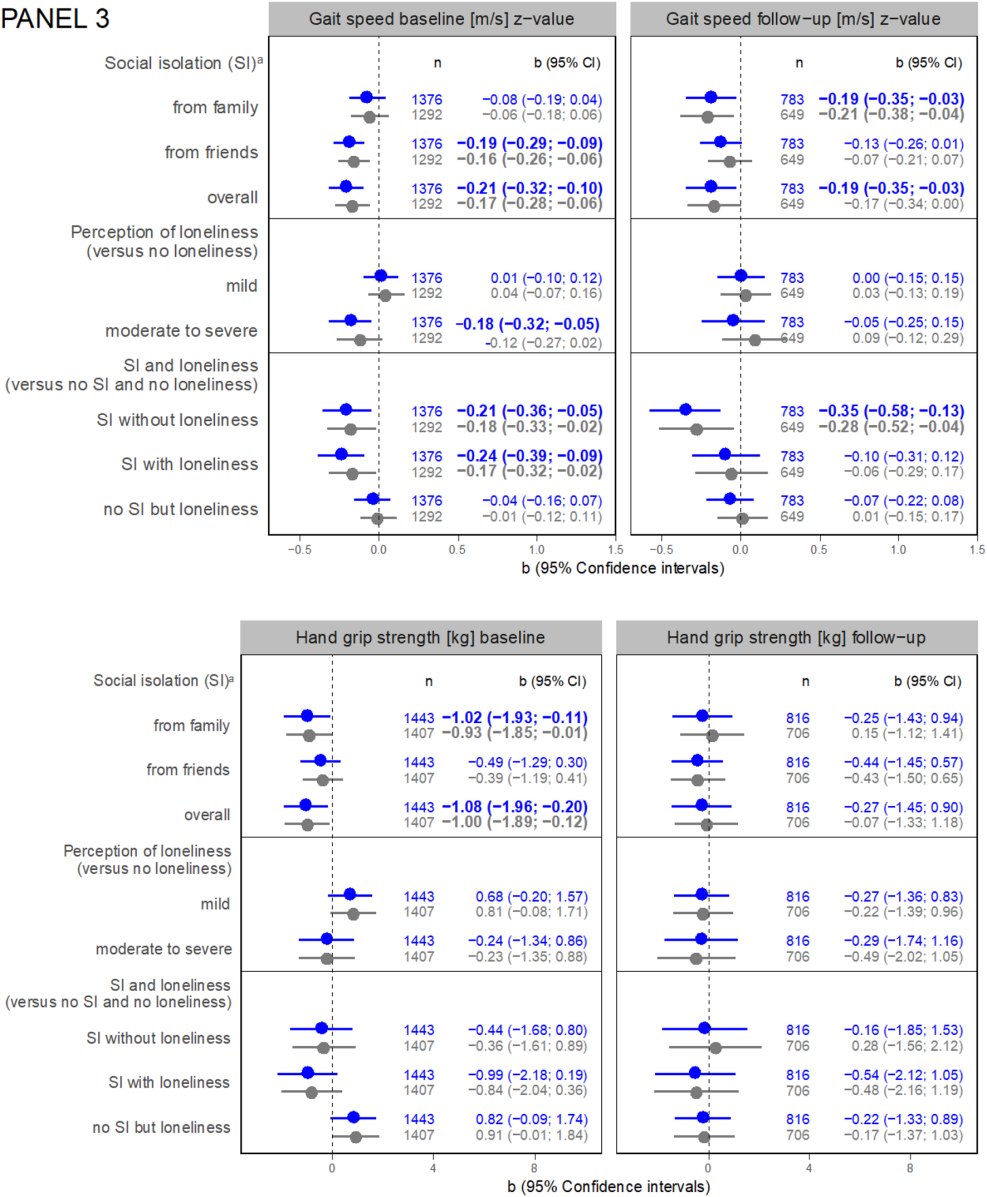
Panel 1: associations between social isolation or loneliness and inflammatory markers (regression coefficients with 95% confidence intervals). a versus not socially isolated from the respective group (i.e. family, friends, or overall); *GFR* Cystatin C based glomerular filtration rate [ml/min], *hs-CRP* high-sensitivity C reactive protein, *IL-6* interleukin 6, *SI* social isolation; IL-6 at follow-up not applicable. Panel 2: associations between social isolation or loneliness and cardiac markers (regression coefficients with 95% confidence intervals). *GDF-15* growth/differentiation factor 15, *NT-proBNP* N-terminal pro-brain natriuretic peptide, *hs-cTnI* high-sensitivity troponin I, *hs-cTnT* high-sensitivity troponin T.Panel 3: associations between social isolation or loneliness and functional parameters (regression coefficients with 95% confidence intervals).

Regarding the results of linear regression analyses, an association between SI from friends and hs-CRP at 3-years FU is shown (b = 0.14, 95% Confidence interval (CI) = 0.04; 0.24) adjusted for age and sex (Model 1) (Fig. 2, Panel 1). This association became not significant after further adjustment (Model 2, b = 0.09, 95% CI = −0.01; 0.20). Moderate to severe perceived loneliness was associated with hs-CRP at baseline (Model 1, b = 0.13, 95% CI = 0.03; 0.23, Model 2, b = 0.11, 95% CI = 0.00; 0.21).

Also, SI from friends was associated with GDF-15 at baseline (Model 1, b = 0.05, 95% CI = 0.01, 0.10, Fig. 2, Panel 2) and at 3-years FU (Model 1, b = 0.07, 95% CI = 0.01; 0.13) adjusted for age and sex. Yet, no association was detected after further adjustment.

In contrast, it was SI from family that showed a positive association with NT-proBNP at baseline (Model 1, b = 0.15, 95% CI = 0.02; 0.27; Model 2, b = 0.14, 95% CI = 0.02; 0.26) and at FU (Model 1, b = 0.15, 95%CI = −0.01; 0.32; Model 2, b = 0.18, 95% CI = 0.01; 0.36). Regression coefficients for the association between total SI baseline and NT-proBNP in FU were higher in SI without loneliness than SI with loneliness (each compared to being not socially isolated and not lonely). SI from friends was associated with higher hs-cTnT at FU in Model 1 (b = 0.12, 95% CI = 0.03; 0.20; Model 2, b = 0.08, 95% CI = −0.01; 0.17). SI without loneliness was more strongly associated with hs-cTnT than SI with loneliness.

Regarding functional parameters, SI from friends (Model 1, b = −0.19, 95% CI = −0.29; −0.09; Model 2, b = −0.16, 95% CI = −0.26; −0.06) and SI overall (Model 1, b = −0.21, 95% CI = −0.32; −0.10; Model 2, b = −0.17, 95% CI = −0.28; −0.06) were linked to lower gait speed at baseline, so was moderate to severe loneliness compared to no loneliness at baseline (Model 1, b = −0.18, 95% CI = −0.32; −0.05 ; Model 2, −0.12, 95% CI = −0.27; 0.02). SI from family was also negatively associated with gait speed at FU (Model 1, b = −0.19, 95% CI = −0.35; −0.03; Model 2, b = −0.21, 95% CI = −0.38; −0.04). For SI overall, the respective estimates were (Model 1, b = −0.19, 95% CI = −0.35; −0.03; Model 2, b = −0.17, 95% CI = −0.34; 0.00). Particularly in FU, SI proves to be a far more important determinant of gait speed than loneliness.

SI from family and overall but not loneliness was cross-sectionally associated with lower hand grip strength in Model 1 and Model 2 (Fig. 2, Panel 3) (SI from family, Model 1, b = −1.02, 95% CI = −1.93; −0.11; Model 2, b = −0.93, 95% CI = −1.85; −0.01, SI overall, Model 1, b = −1.08, 95% CI = −1.96; −0.20; Model 2, b = −1.00, 95% CI = −1.89; −0.12) compared to the non-exposed counterparts. There is even a tendency that mild loneliness is associated with better hand grip strength at baseline compared to no loneliness.

SI from family or SI from friends were not associated with survival, but the overall index was (Hazard ratio HR 1.39 (1.15; 1.67)) (Table 2, Model 2). SI without and with loneliness were similarly associated with higher mortality (compared to not being socially isolated or lonely) with HR 1.48 (1.12; 1.94) and 1.45 (1.21; 1.87), each Model 2, respectively.

**Table 2:** Association of loneliness and social isolation with mortality. Results of Cox proportional hazards models, Hazard ratio (95% CI)

Predictor	Deaths (n = 527)	Model 1 Adjusted for age and sex	Model 2 Adjusted for age, sex, education, living alone, number of medications, BMI, smoking, alcohol, and GFR at baseline
		Hazard ratio (95% CI)	Hazard ratio (95% CI)
SI from family baseline (Ref: no SI from family)	143	1.21 (0.99; 1.46)	1.22 (1.00; 1.49)
SI from friends baseline (Ref: no SI from friends)	258	1.15 (0.96; 1.36)	1.13 (0.94; 1.35)
SI overall baseline (Ref: no SI overall)	180	**1.31 (1.10; 1.58)**	**1.39 (1.15; 1.67)**
Loneliness baseline			
None	271	Ref	Ref
Mild loneliness	150	1.05 (0.86; 1.29)	1.02 (0.83; 1.25)
Moderate to severe loneliness	106	**1.27 (1.01; 1.59)**	1.26 (1.00; 1.59)
SI and loneliness			
No SI, no loneliness	189	Ref	Ref
SI without loneliness	82	**1.40 (1.08; 1.82)**	**1.48 (1.12; 1.94)**
SI with loneliness	98	**1.40 (1.10; 1.80)**	**1.45 (1.21; 1.87)**
No SI but loneliness	158	1.16 (0.94; 1.43)	1.12 (0.90; 1.40)

Bold letters indicate significance at p < 0.05

*BMI* body mass index, *CI* Confidence interval, *GFR* Cystatin C based glomerular filtration rate [ml/min], *Ref.* Reference, *SI* Social isolation

## Discussion

In this medium-sized cohort study including older adults aged 65 + years, the identified prevalence for SI overall of 26.7% was similar to that found in previous literature [6, 12]. With a prevalence of 28.8% of mild loneliness and 16.1% of moderate to severe loneliness, our prevalence seems higher compared to the literature [4]. Notably, the age- and sex-adjusted correlation of SI and, less clearly, also loneliness was seen especially with functional parameters such as gait speed and hand grip strength. Furthermore, we observed statistically significant adverse associations for SI (particularly from friends) and some biomarkers: hs-CRP, GDF-15, and hs-cTnT. SI but not loneliness was associated with 10-year mortality (in the fully adjusted model).

### Inflammation

We observed higher hs-CRP levels in socially isolated older adults at follow-up, however, SI was not statistically significantly associated with hs-CRP in the fully adjusted model. Therefore, our study is in line with the review of Smith and colleagues [33] who showed a significant association between SI and CRP, however, not in most-adjusted analyses. Interestingly, in contrast to most other biomarkers we analyzed, loneliness seemed to be associated with hs-CRP cross-sectionally.

In general, the association between SI and loneliness with inflammatory parameters is conflicting. The following objections may explain the divergent results, which also apply to the other biomarkers analyzed: First, chronicity of SI or loneliness may modify the associations [34]. For instance, Guo and colleagues have recently shown higher HR for incident cardiovascular diseases (CVD) in fluctuating (HR = 1.27, 95% CI = 1.01;1.59) and more pronounced in consistently high SI (HR = 1.45, 95% CI = 1.13; 1.85) compared to low SI [35]. Second, different exposure measurements and follow-up periods may have contributed to inconsistent results.

### Cardiac markers

SI and loneliness were associated with an increased risk for coronary heart disease and stroke (see review [10]), CVD mortality (see review [36]), and heart failure [11, 37]. Studies have shown a predictive role of GDF-15 across an array of CVD including chronic heart failure [38]. Also, NT-proBNP has proven useful as indicator of myocardial damage. However, a recent study analyzing the association between NT-proBNP and loneliness did not find an association [39], despite a known link of GDF-15 with psychosocial stress and mental health, e.g. [21, 22, 40]. At baseline and FU, we saw positive associations between SI from friends and GDF-15 in model 1. For the particular role of SI from friends as opposed to family, the following reason might be considered: Friendships are chosen freely and may therefore exert stronger direct or buffer effects against social stress than interactions with family. Nevertheless, additional research that allows to distinguish marital status from other kinship (both included in the Lubben subscale family) would be helpful. As we adjusted for living alone, further adjustment for married yes/no did not seem appropriate but might be done in future studies. It can additionally be presumed that interactions with friends are associated with increased PA (in terms of the necessity to leave the house) [41]. A negative association between SI or loneliness and PA has been confirmed (see Lindsay Smith 2017 for a review [42]). More recently, associations between SI and objectively measured activity in a study conducted in the UK were found. Thereby, time spent in sedentary behaviour was higher in isolated participants while PA was less frequent. Interestingly, loneliness was not associated with PA or sedentary behaviour [43]. Moreover, the available evidence suggests an interaction between systemic inflammation and psychosocial stress in the association with hs-cTnT in patients with coronary heart disease [44]. Corroboration of our descriptive results would help to confirm our findings on SI and hs-cTnT.

### Functional parameters

The relationship between SI and reduced PA might also partly explain the inverse association between SI and gait speed which we discovered in line with the work of Merchant and colleagues [23]. We have additionally found negative associations between moderate to severe loneliness (versus no loneliness) and gait speed at baseline. However, the association between loneliness and reduced gait speed may be mediated by depression [45] as loneliness and depression are highly correlated (Pearson`s r = 0.47, p < 0.001 in our analyses) and a common symptom of depression is psychomotor retardation.

Cross-sectionally, we revealed a negative association for SI from family (and SI overall) with hand grip strength. Evidence shows that hand grip strength serves as an explanatory factor of overall strength, bone mineral density, fractures, and falls among others [46]. Chronic loneliness was associated with a persistent decline in hand grip strength in older adults but more pronounced in men than in women [47].

### Mortality

The association between SI, loneliness and all-cause mortality appears to be robust in the literature [9, 14, 15] and is confirmed by our analyses for SI overall. A recently published article summarized that evidence on mortality is consistent across causes of death, countries, and sex but research is still needed on e.g. marginalized groups and modes of socializing [48]. Moreover, we assume that the biological mechanisms linking SI to mortality need to be further elucidated.

### Loneliness versus social isolation

Comparing the relative impact of SI and loneliness on health, Hong and colleagues [49] argued that SI was more predictive of mortality and loneliness was a stronger predictor of psychological outcomes. Also, the study of Smith and Victor for example where the authors compared associations between different types of lonely/isolated participants and physical/mental health outcomes pointed in the same direction [50]. The adjusted Odds ratio (OR) of experiencing high depressive symptoms was OR = 6.60, 95% CI = 5.05; 8.63 in moderately lonely participants and OR = 2.29, 95% CI = 1.49, 3.53 in moderate SI each compared to no/low loneliness and social isolation. For two or more chronic conditions, the respective values were OR = 1.89, 95% CI = 1.56; 2.28, OR = 1.20, 95% CI = 0.91, 1.57.

Similarly, when comparing subjective loneliness and network quality versus network size or living alone, loneliness predicted mental health while network size and living alone predicted physical and cognitive health [51]. Cornwell and Waite found that the association between SI or loneliness and mental health was supported by a strong association between loneliness and mental health and disappeared when loneliness was excluded from their models [52]. In contrast, after adjusting statistically for demographic factors and baseline health, social isolation remained significantly associated with mortality (HR = 1.26, 95% CI = 1.08, 1.48), but loneliness did not (HR = 0.92, 95% CI = 0.78, 1.09) in a study conducted by Steptoe and colleagues [53]. Nevertheless, Holt-Lunstad and colleagues concluded that studies including several measures simultaneously – a prerequisite for a comparison of the estimates – are rare [8].

### Limitations

Even though the ActiFE-cohort was intended to be a representative cohort of community-dwelling older adults, participation at baseline was relatively low and the observed health status was above average. Although a comparison of baseline population and follow-up population suggested an under-representation of the socially isolated and lonely population in follow-up, the proportion of elderly perceiving loneliness was higher than what has been reported [4]. More importantly, it is argued that single-item measures which we used for assessing loneliness are (i) unambiguous and narrow in scope (for a discussion see e.g. [54]). (ii) The prevalence of loneliness may differ depending on the wording of the question used. For example, higher levels of loneliness were shown in women when using a more direct measure (naming “loneliness”), while loneliness was more prevalent in men when using an indirect measure potentially because of e.g. social stigmatization (discussed in [55]). In their article, Mund and colleagues [56] concluded based on a comparative research, that loneliness can be measured using single items where the use of multi-item scales is not possible due to financial or time constraints [56]. However, the authors emphasized that different dimensions (emotional and social loneliness) cannot be distinguished adequately by one question. The use of this single question might also be the reason why results were clearer with the validated social isolation assessment in our analyses.

Furthermore, we used logarithmic transformation, which is common for biological parameters and ensures the validity of the analysis but can make interpretation difficult while necessary due to skewed distribution. Another important limitation is that the associations we show are small. Our analysis was intended to be descriptive rather than confirmatory as we focused on exploring patterns. Therefore, our results should be verified by other studies. Furthermore, reverse causality has also to be considered and we did not adjust for marital status but instead for living alone.

## Conclusion and implications

Social isolation, to a lesser extend loneliness, is consistently associated with decreased functional parameters such as gait speed and handgrip strength and a high risk for 10-year mortality. With respect to biomarkers SI was partly associated with high hs-CRP, NT-proBNP, and hs-cTnT levels; loneliness with high hs-CRP levels.

## Supplementary Information

Below is the link to the electronic supplementary material.

Supplementary file1 (PDF 165 KB)

## Data Availability

You can obtain the data and code through personal contact with the corresponding author: stefanie.braig@uni-ulm.de.
