# Supplementary material for: Social isolation, loneliness and the relationship with serum biomarkers, functional parameters and mortality in older adults
Source: Aging Clin Exp Res. 2025 May 3;37(1):140. doi: 10.1007/s40520-025-03041-4 (PMC12049398; doi:10.1007/s40520-025-03041-4)
Supplement: Supplementary file 1 — Supplementary file1 (PDF 165 KB) [file 40520_2025_3041_MOESM1_ESM.pdf]

Social isolation, loneliness and the relationship with serum biomarkers, functional parameters and mortality in older adults; Aging Clinical and Experimental Research;
Stefanie Braig, Michael D. Denkinger, Dhayana Dallmeier, Jochen Klenk, Dietrich Rothenbacher for the ActiFE Study Group. Corresponding author: Stefanie Braig, Institute of Epidemiology and Medical Biometry, Ulm University, Ulm, Germany, stefanie.braig@uni-ulm.de

Online resource 1: Outcome measurements

**Variables**	**Unit**	**Method**	**Further information**
**Baseline**			
high-sensitivity C reactive protein (hs-CRP)	mg/l	Immunonephelometery on a BNA II (Siemens Eschborn, Germany)	Inter assay coefficient of variation (CV) 5.2–6.4%
Interleukin-6 (IL-6))	pg/ml	ELISA Quantikine Human IL-6 Immunoassay (R&D Systems)	Range 0.156-10 pg/ml
growth differentiation factor-15 (GDF-15)	pg/ml	ElectroChemiLuminescenceImmunoassay (ECLIA), Roche Diagnostics, Mannheim, Germany, Cobas Elecsys 411	CV <5%, Lower limit of detection (LOD)=10 ng/l, measuring range 27.6–12,700 ng/l.
N-terminal pro-brain natriuretic peptide (NT-proBNP)	pg/ml	ECLIA, Elecsys NT-proBNP II Test, 2010; Roche Diagnostics, Mannheim, Germany	CV <5 %, lower LOD 5 pg/ml
high-sensitivity troponin I (hs-cTnI)	ng/l	ARCHITECT STAT, Abbott Diagnostics	Within-laboratory imprecision of ≤10%. CV across the range of 10 to 50,000 ng/L (LOD <2.0 ng/l), within-run and within laboratory CV of <5.5%.
high-sensitivity troponin T (hs-cTnT)	ng/l	Cobas E 2010	LOD=5 ng/l, inter assay CV 4.965.43%
high-sensitivity cystatin C (hs-cystatin C)	mg/l	Nephelometrie on a BNA II (Siemens Eschborn, Germany)	Measuring range: 0.05–7.25
**Follow-up**			
Hs-CRP	mg/l	Immunonephelometery on a BNA II (Siemens Eschborn, Germany)	CV 1.96–2.37%
GDF-15	pg/ml	ECLIA, Cobas e411 Roche Diagnostics, Mannheim, Germany	Measuring range 364-20000 pg/ml, CV 3.2%-2.46%
NT-proBNP	pg/ml	ECLIA, Cobas e411 Roche Diagnostics, Mannheim, Germany	Inter assay CV 4.57–5.39%
hs-cTnI	ng/l	Chemiluminescent MicroparticleImmunoassay (CMIA), Abott Diagnostics	CV 4.25–9.11%
hs-cTnT	ng/l	COBAS e411 Roche Diagnostics, Mannheim, Germany	CV 1.8–7.78%

Online resource 2:

Median (interquartile range (Q1; Q3)) of specific biomarkers and Spearman correlation coefficients between biomarkers and measures of loneliness and social isolation partially adjusted for age and sex

	Median (Q1; Q3)	n	Social isolation from family BL continuously measured	Social isolation from friends BL continuously measured	Social isolation overall BL continuously measured	Loneliness BL continuously measured
**Inflammatory markers**						
hs-CRP BL (mg/l)	1.7 (0.9; 3.7)	1435	-0.02, p=0.48	0.05, p=0.072	0.03, p=0.34	0.04, p=0.15
hs-CRP FU (mg/l)	1.4 (0.7; 3.0)	805	0.01, p=0.98	**0.08, p=0.018**	0.06, p=0.10	0.01, p=0.81
IL-6 BL BL[a] (pg/ml)	1.9 (1.4; 3.0)	1438	-0.01, p=0.82	0.03, p=0.31	0.02, p=0.42	0.03, p=0.34
**Cardiac markers**						
GDF-15 BL (pg/ml)	1394.0 (1034.0; 1948.0)	1431	0.01, p=0.69	**0.05, p=0.043**	0.04, p=0.13	0.03, p=0.31
GDF-15 FU (pg/ml)	1406.0 (1052.0; 1965.0)	806	0.00, p=0.90	0.07, p=0.060	0.05, p=0.16	0.03, p=0.42
NT-proBNP BL (pg/ml)	152.0 (81.5; 318.0)	1435	0.03, p=0.28	0.01, p=0.76	0.03, p=0.31	0.00, p=0.97
NT-proBNP FU (pg/ml)	161.1 (87.6; 362.3)	805	0.05, p=0.17	0.04, p=0.21	0.06, p=0.10	-0.01, p=0.84
Hs-cTnT BL (ng/l)	2.5 (2.5; 10.2)	1435	0.01, p=0.67	**0.06, p=0.028**	**0.05, p= 0.045**	0.00, p=0.89
Hs-cTnT FU (ng/l)	8.9 (5.4; 14.6)	805	0.00, p=0.90	**0.11, p=0.002**	**0.08, p=0.016**	-0.00, p=0.95
Hs-cTnI BL (ng/l)	5.9 (4.4; 9.1)	1434	-0.02, p=0.39	-0.01, p=0.70	-0.01, p=0.72	0.00, p=0.94
Hs-cTnI FU (ng/l)	5.2 (3.9; 7.7)	788	0.00, p=0.98	0.02, p=0.43	0.03, p=0.46	-0.01, p=0.79
**Functional parameters**						
Gait speed (m/s) BL z-score	-0.19 (-0.7; 0.4)	1376	**-0.10, p<0.001**	**-0.13, p<0.001**	**-0.14, p<0.001**	**-0.06, p=0.019**
Gait speed (m/s) FU z-score	-0.02 (-0.8, 0.7)	783	**-0.09, p=0.007**	**-0.11, p=0.003**	**-0.12, p<0.001**	-0.09, p=0.80
Hand grip strength (kg) BL, mean (SD)	32.0 (11.0)	1443	**-0.06, p=0.034**	**-0.06, p=0.032**	**-0.07, p=0.004**	0.01, p=0.63
Hand grip strength (kg) FU, mean (SD)	28.7 (10.6)	816	-0.04, p=0.24	**-0,07, p=0.040**	**-0.08, p=0.032**	-0.02, p=0.51

BL: Baseline, FU: Follow-up, GDF-15: Growth/differentiation factor 15, hs-CRP: C reactive protein, hs-cTnI: high-sensitive troponin I, hs-cTnT: high-sensitive troponin T, IL-6: Interleukin 6, NT-proBNP: N-terminal pro-brain natriuretic peptide, Q1: 1st Quartile, Q3: 3th Quartile, SD: Standard deviation, a: only available at baseline, bold letters indicate statistical significance at p<0.05

(…continued): Point serial correlation coefficients between biomarkers and measures of social isolation and loneliness partially adjusted for age and sex

	n	No SI and no loneliness	n	SI without loneliness	n	SI with loneliness	n	No SI but loneliness
**Inflammatory markers**								
hs-CRP BL (mg/l)	608	-0.04, p=0.12	182	-0.00, p=0.88	201	0.05, p=0.07	444	0.01, p=0.68
hs-CRP FU (mg/l)	376	-0.01, p=0.88	80	0.03, p=0.40	93	0.03, p=0.32	256	-0.04, p=0.29
IL-6 BL BL[a](pg/ml)	609	-0.02, p=0.53	183	-0.01, p=0.72	201	0.03, p=0.34	445	0.01, p=0.84
**Cardiac markers**								
GDF-15 BL (pg/ml)	606	-0.04, p=0.17	181	0.04, p=0.13	200	0.00, p=0.95	444	0.01, p=0.75
GDF-15 FU (pg/ml)	377	-0.00, p=0.88	80	0.03, p=0.47	93	0.00, p=0.95	256	-0.01, p=0.73
NT-proBNP BL (pg/ml)	608	-0.04, p=0.11	182	0.04, p=0.069	201	0.02, p=0.54	444	-0.00, p=0.94
NT-proBNP FU (pg/ml)	376	0.01, p=0.79	80	0.04, p=0.23	93	0.00, p=0.94	256	-0.04, p=0.32
Hs-cTnT BL (ng/l)	608	-0.04, p=0.090	182	0.03, p=0.24	201	0.01, p= 0.82	444	0.02, p=0.43
Hs-cTnT FU (ng/l)	376	0.01, p=0.73	80	0.05, p=0.16	93	-0.01, p=0.69	256	-0.04, p=0.32
Hs-cTnI BL (ng/l)	607	0.04, p=0.24	182	-0.02, p=0.57	201	-0.00, p=0.92	444	-0.02, p=0.46
Hs-cTnI FU (ng/l)	368	0.03, p=0.41	76	0.02, p=0.64	92	-0.02, p=0.53	252	-0.03, p=0.46
**Functional parameters**								
Gait speed (m/s) BL z-score	591	**0.07, p=0.012**	171	**-0.05, p=0.043**	190	**-0.07, p=0.007**	424	0.02, p=0.44
Gait speed (m/s) FU z-score	366	0.07, p=0.052	79	**-0.10, p=0.005**	89	-0.01, p=0.77	249	-0.00, p=0.96
Hand grip strength (kg) BL	616	-0.00, p=0.79	181	-0.02, p=0.34	204	**-0.06 p=0.031**	442	**0.07, p=0.001**
Hand grip strength (kg) FU	380	0.02, p=0.57	81	-0.00, p=0.99	95	-0.02, p=0.57	260	-0.00, p=0.84

BL: Baseline, FU: Follow-up, GDF-15: Growth/differentiation factor 15, hs-CRP: C reactive protein, hs-cTnI: high-sensitive troponin I, hs-cTnT: high-sensitive troponin T, IL-6: Interleukin 6, NT-proBNP: N-terminal pro-brain natriuretic peptide, Q1: 1st Quartile, Q3: 3th Quartile, SD: Standard deviation, a: only available at baseline, bold letters indicate statistical significance at p<0.05

Online resource 3:

Median (interquartile range (Q1; Q3)) of specific biomarkers and Spearman correlation coefficients between biomarkers and measures of loneliness and social isolation partially adjusted for age and sex

Data set is reduced on participants with follow up

	Median (Q1; Q3)	n	Social isolation from family BL continuously measured	Social isolation from friends BL continuously measured	Social isolation overall BL continuously measured	Loneliness BL continuously measured
**Inflammatory markers**						
hs-CRP BL (mg/l)	1.6 (0.8; 3.3)	804	-0.02, p=0.55	0.03, p=0.48	0.01, p=0.88	**0.08, p=0.018**
IL-6 BL BL[a] (pg/ml)	1.8 (1.3; 2.6)	806	-0.02, p=0.55	0.02, p=0.57	0.01, p=0.85	0.03, p=0.47
**Cardiac markers**						
GDF-15 BL (pg/ml)	1302.0 (987.2; 1746.0)	801	0.00, p=0.99	0.06, p=0.080	0.04, p=0.25	0.05, p=0.16
NT-proBNP BL (pg/ml)	132.5 (73.2; 251.5)	804	0.04, p=0.32	0.04, p=0.30	0.05, p=0.14	-0.01, p=0.83
Hs-cTnT BL (ng/l)	2.5 (2.5; 8.3)	804	0.01, p=0.78	0.06, p=0.10	0.05, p= 0.14	-0.00, p=0.95
Hs-cTnI BL (ng/l)	5.7 (4.3; 8.3)	803	-0.04, p=0.26	-0.01, p=0.79	-0.02, p=0.60	0.01, p=0.77
**Functional parameters**						
Gait speed (m/s) BL z-score, mean (SD)	0.2 (1.0)	778	**-0.13, p<0.001**	**-0.14, p<0.001**	**-0.17, p<0.001**	-0.05, p=0.16
Hand grip strength (kg) BL, mean (SD)	33.5 (11.0)	814	-0.04, p=0.29	-0.06, p=0.12	**-0.07 p=0.048**	0.03, p=0.43

BL: Baseline, FU: Follow-up, GDF-15: Growth/differentiation factor 15, hs-CRP: C reactive protein, hs-cTnI: high-sensitive troponin I, hs-cTnT: high-sensitive troponin T, IL-6: Interleukin 6, NT-proBNP: N-terminal pro-brain natriuretic peptide, Q1: 1st Quartile, Q3: 3th Quartile, SD: Standard deviation, a: only available at baseline bold letters indicate statistical significance at p<0.05

(…continued): Point serial correlation coefficients between biomarkers and measures of social isolation and loneliness partially adjusted for age and sex

Data set is reduced on adults that where followed-up

	n	No SI and no loneliness	n	SI without loneliness	n	SI with loneliness	n	No SI but loneliness
**Inflammatory markers**								
hs-CRP BL (mg/l)	374	**-0.07, p=0.04**	81	-0.00, p=0.86	92	0.03, p=0.38	257	0.05, p=0.15
IL-6 BL BL[a] (pg/ml)	375	0.01, p=0.82	82	-0.02, p=0.57	92	-0.02, p=0.54	257	0.02, p=0.59
**Cardiac markers**								
GDF-15 BL (pg/ml)	372	-0.03, p=0.41	81	0.01, p=0.76	91	0.00, p=0.91	257	0.02, p=0.55
NT-proBNP BL (pg/ml)	375	-0.06, p=0.067	82	**0.09, p=0.015**	92	0.02, p=0.65	257	0.00, p=0.94
Hs-cTnT BL (ng/l)	374	-0.05, p=0.16	81	0.04, p=0.23	92	-0.02, p= 0.62	257	0.04, p=0.29
Hs-cTnI BL (ng/l)	375	0.04, p=0.27	82	-0.00, p=0.98	92	-0.02, p=0.61	257	-0.03, p=0.34
**Functional parameters**								
Gait speed (m/s) BL z-score	364	0.07, p=0.060	77	-0.06, p=0.073	90	-0.07, p=0.059	247	0.01, p=0.66
Hand grip strength (kg) BL	381	-0.02, p=0.49	81	-0.01, p=0.69	95	-0.05 p=0.17	257	0.07, p=0.050

BL: Baseline, FU: Follow-up, GDF-15: Growth/differentiation factor 15, hs-CRP: C reactive protein, hs-cTnI: high-sensitive troponin I, hs-cTnT: high-sensitive troponin T, IL-6: Interleukin 6, NT-proBNP: N-terminal pro-brain natriuretic peptide, Q1: 1st Quartile, Q3: 3th Quartile, SD: Standard deviation, a: only available at baseline, bold letters indicate statistical significance at p<0.05
